# A Potential Role for Common Mycorrhizal Networks (CMNs) in Mediating Response Strategies and Signaling Between Different Plant Combinations Under Salt Stress

**DOI:** 10.3390/jof12040242

**Published:** 2026-03-26

**Authors:** Jingwen Zheng, Qingyun Liu, Xueying Yang, Yongxue Xie, Zetong Gao, Xiaodong Ma

**Affiliations:** Xinjiang Key Laboratory of Special Species Conservation and Regulatory Biology, College of Life Sciences, Xinjiang Normal University, Urumqi 830017, China; jingwenz10@126.com (J.Z.); lqy1811@126.com (Q.L.); yxy20030113@126.com (X.Y.); m13999125934@126.com (Y.X.); 18829818672@163.com (Z.G.)

**Keywords:** common mycorrhizal networks, salt stress, plant hormones, nitrogen transfer, physiological responses, *Glycyrrhiza inflata*, *Lycium ruthenicum*

## Abstract

Soil salinization is one of the main stress factors limiting plant growth and ecosystem restoration in arid regions. Arbuscular mycorrhizal fungi (AMF) can form common mycorrhizal networks (CMNs) that potentially facilitate resource and signal exchange between plants. In this study, we investigated whether such processes associated with AMF connectivity might contribute to salt tolerance in different plant combinations, using *Glycyrrhiza inflata* and *Lycium ruthenicum*. However, under salt stress, it remains unclear how different plant combinations (conspecific vs. heterospecific) may differentially benefit from CMN-mediated processes under salt stress, and whether such processes involve coordinated stress signaling and nitrogen transfer. This study used *Glycyrrhiza inflata* (a leguminous N-fixing plant with a “N-input” strategy) and *Lycium ruthenicum* (a deep-rooted desert shrub with a “resource-use efficiency” strategy) as materials to construct conspecific and heterospecific plant combinations: G-G (*G. inflata*-*G. inflata*), L-L (*L. ruthenicum*-*L. ruthenicum*), G-L (*G. inflata*-*L. ruthenicum*), and L-G (*L. ruthenicum*-*G. inflata*). Four salt stress levels were set (NaCl concentrations of 0, 150, 250, and 350 mmol·L^−1^), along with AMF inoculation treatments. The study evaluated responses in AMF colonization, nitrogen transfer, biomass, root structure, photosynthetic characteristics, antioxidant capacity, osmotic regulation, and hormone levels. The results show that: (1) AMF colonization rates in all inoculated groups significantly decreased with increasing salt concentration, with the G-L combination showing a smaller decline; (2) The G-G combination maintained strong root activity and photosystem stability under high salt stress, exhibiting higher salt tolerance; (3) In conspecific combinations, the JA-Pro signaling pathway was dominant, whereas in heterospecific combinations, the ABA-SOD pathway prevailed, indicating differences in hormone regulation mechanisms among different combinations; (4) ^15^N transfer efficiency was significantly higher in conspecific combinations than in heterospecific combinations (*p* < 0.05), and increasing salt concentrations limited the resource-sharing ability of heterospecific combinations. In summary, our results revealed distinct physiological and hormonal responses in conspecific versus heterospecific plant combinations under salt stress when grown in an AMF-colonized system that permits hyphal connections. These patterns were consistent with a potential role of CMNs in signal coordination and resource sharing, although further experiments with disrupted hyphal connections would be required to confirm this mechanism.

## 1. Introduction

Soil salinization is one of the most destructive abiotic stresses in arid and semi-arid regions. It severely restricts plant growth and vegetation ecological restoration by disrupting plant ion balance, inducing oxidative damage, and inhibiting photosynthesis and nutrient uptake [1,2,3]. In typical arid riparian ecosystems such as the lower reaches of the Tarim River, the lower reaches of the Colorado River in the southwestern United States, and the inland arid river regions of Australia, high evaporation rates trigger capillary rise of groundwater salinity, further exacerbating soil salinization. At the same time, the arid environment suppresses soil microbial nitrogen mineralization, combined with nitrogen volatilization and leaching, leading to extreme soil nitrogen deficiency, forming a dual constraint of ‘salt stress–nitrogen deficiency,’ which becomes the central bottleneck for vegetation restoration [4,5,6,7]. Unlike phosphorus, which is largely insoluble, poorly mobile [8], and accessible to plants only after solubilization or via mycorrhizal hyphae [9,10], nitrogen—despite being easily leachable—can be rapidly taken up by plants when available, and its supply often imposes a more immediate constraint on plant community restoration in this area. Therefore, the efficiency of nitrogen transfer and its allocation strategy are key factors determining the construction and restoration potential of plant communities in this area.

Arbuscular mycorrhizal fungi (AMF), as widely occurring soil microorganisms, can form symbiotic relationships with the vast majority of terrestrial plants, enhancing plant salt tolerance through various means such as improving host ion homeostasis, activating antioxidant systems, coordinating hormone signals, and increasing photosynthetic efficiency [11,12,13,14,15,16]. When AMF hyphae connect the roots of different plants, they form common mycorrhizal networks (CMNs). This “underground interconnected network” not only mediates the transfer and distribution of nutrients such as nitrogen and phosphorus between plants, but also transmits stress signals, allowing unstressed plants to activate their defense mechanisms in advance, known as the “priming effect,” playing a key role in plant interactions and community stability [17,18,19,20]. Existing studies have confirmed that CMNs can promote nutrient sharing and mutualistic symbiosis among plants [21,22,23,24,25,26,27,28,29,30], but their functions are jointly regulated by plant phylogenetic relationships, carbon allocation strategies, and types of stress. Currently, research on CMNs has mostly focused on the stress responses of individual plants, whereas under salt stress, the modes of signal transmission, nitrogen transfer efficiency, and physiological adaptation mechanisms mediated by CMNs among different plant combinations (same species/different species) remain inadequately explained.

Plant hormones (abscisic acid, ABA; jasmonic acid, JA; and salicylic acid, SA) are core signaling molecules in stress responses. Soil nitrogen deficiency can induce root architecture remodeling and affect photosynthetic system function. Both factors trigger the upregulation of the ABA signaling pathway, suggesting a close connection between nutrient status and hormone signaling [23]. Although nitrogen transfer mediated by CMNs has been shown to help maintain plant growth under stress [22], several key questions remain unresolved. It remains unclear how the CMNs coordinate nitrogen sharing and hormone signaling under salt stress, and whether different plant combinations (conspecific/heterospecific) form distinct signaling response patterns via CMNs, thereby determining the specificity of their salt tolerance strategies. Answering these questions has become a central entry point for revealing the ecological functions of CMNs and the mechanisms of plant stress adaptation.

*Glycyrrhiza inflata* and *Lycium ruthenicum* are typical mycorrhizal-dependent plants in the lower reaches of the Tarim River, and there are significant differences in their ecological niches and nutrient acquisition strategies: *Glycyrrhiza inflata,* a leguminous nitrogen-fixing plant, relies on symbiosis with root nodule bacteria and AMF to achieve nitrogen input, and is considered a “nitrogen-input” species; *Lycium ruthenicum,* a desert shrub, efficiently utilizes limited resources through its deep roots and strong salt tolerance, and is considered a “resource-use-efficiency” species [24,25,26]. This functional difference makes it an ideal research system for investigating how CMNs mediate responses of different plant combinations under salt stress.

Therefore, this study takes *Glycyrrhiza inflata* and *Lycium ruthenicum* as the research subjects, sets up four plant combinations (including same-species and mixed-species combinations), and designs three salt concentration gradient treatments. Through pot experiments, it focuses on exploring how CMNs mediate the transfer and responses of key signaling substances such as ABA, JA, and SA between donor and receiver plants. The study systematically analyzes the effects of salt stress on the growth, physiological characteristics, endogenous hormones, and nitrogen content of two plant species. It aims to clarify: (1) whether conspecific and heterospecific plant combinations exhibit systematic differences in hormone signal response strategies mediated by CMNs; (2) the relationship between these signal response differences and nitrogen transfer efficiency, as well as the coupling of physiological processes such as photosynthesis, antioxidant activity, and osmotic regulation, and their overall regulatory effects on plant salt stress adaptation. This research aims to explore, from a “signal-nutrient-physiology” perspective, how AMF-colonized plants might interact via shared mycorrhizal networks under salt stress, and to infer the possible role of CMNs in regulating plant interactions and systemic adaptation.

Based on the above rationale, we proposed the following hypotheses:

(1) Phylogenetic relatedness enhances CMNs functionality—conspecific plant combinations exhibit greater CMNs stability, more efficient nitrogen transfer, and stronger stress signal synchronization than heterospecific combinations under salt stress;
(2) Hormonal signaling pathways are combination-dependent—conspecific combinations predominantly rely on the JA-Pro pathway to coordinate osmotic adjustment and defense, whereas heterospecific combinations preferentially activate the ABA-SOD pathway to mitigate oxidative damage;
(3) Signal–nutrient–physiology coupling determines salt adaptation—the divergence in hormonal signaling between conspecific and heterospecific combinations is functionally coupled with differences in nitrogen transfer efficiency and photosynthetic/antioxidant capacity, collectively shaping their distinct salt tolerance strategies.

To test these hypotheses, we conducted a pot experiment using four plant combinations (*Glycyrrhiza inflata*-*Glycyrrhiza inflata*, *Lycium ruthenicum*-*Lycium ruthenicum*, *Glycyrrhiza inflata*-*Lycium ruthenicum*, *Lycium ruthenicum*-*Glycyrrhiza inflata*) under four salt levels with AMF inoculation, and systematically analyzed CMNs-mediated signal transfer, nitrogen sharing, and multi-level physiological responses.

## 2. Materials and Methods

### 2.1. Plants and Fungal Inocula

The tested *Glycyrrhiza inflata* seeds, *Lycium ruthenicum* seeds, and river sand were all collected from the banks of the lower reaches of the Tarim River. Full and plump *Glycyrrhiza inflata* seeds were selected and their seed coats were damaged with a file for later use. *Lycium ruthenicum* seeds were pre-germinated in a 40 °C water bath for 48 h. After germination, they, along with the *Glycyrrhiza inflata* seeds, were disinfected with 75% ethanol for 15 min, then rinsed with clean water and set aside.

*Funneliformis mosseae* (BGC XJ01) was used as the AMF in this study and was provided by the Microbial Laboratory of Plant Nutrition and Resources Research Institute, Beijing Academy of Agricultural and Forestry Sciences. The fungal mixture contains 25 spores per gram. The fungal inoculum consists of a mixture of soil, spores, hyphae, and mycorrhizal fragments. The inoculation process involved the addition of 20 g of the inoculum mixture each time.

The growth substrate was composed of river sand and vermiculite. After the river sand was sieved through a 2 mm mesh to remove impurities, it was mixed evenly with vermiculite at a volume ratio of 1:1. The mixture was then sterilized by continuous wet heat at 110 °C and 0.14 MPa for 1 h and set aside for use. Each pot was watered twice a week with distilled water, using the same amount of water each time.

### 2.2. Experimental Device Design

**Figure 1:**
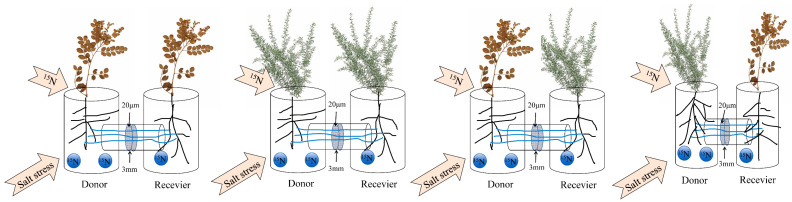
Experimental apparatus.

Two PVC T-shaped pipes were separated by a 3 mm thick PVC ring, forming an air gap; stainless steel screens with a pore size of 20 μm were fixed at both ends of the connecting pipe openings (the 20 μm mesh allowed hyphae to pass through but prevented root passage), and the connection was then secured with hose clamps. The left side of the device served as the donor chamber, and the right side served as the recipient chamber (Figure 1).

### 2.3. Experimental Design, Biological Treatments, and Growth Conditions

The experimental design included four plant combinations: G-G (*Glycyrrhiza inflata*-*Glycyrrhiza inflata*), L-L (*Lycium ruthenicum*-*Lycium ruthenicum*), G-L (*Glycyrrhiza inflata*-*Lycium ruthenicum*), and L-G (*Lycium ruthenicum*-*Glycyrrhiza inflata*). Each combination had five treatments, with three replicates per treatment. All treatments involved AMF inoculation, and salt stress was applied only to the donor plants to investigate the synchronized response effect of stress signaling substances under mycorrhizal connection. The five treatments were as follows (Table 1): CK: blank control, no AMF inoculation, no salt stress, and with physical separation preventing CMNs formation; S0: AMF inoculation, no salt stress; S1: AMF inoculation, 150 mmol·L^−1^ NaCl stress; S2: AMF inoculation, 250 mmol·L^−1^ NaCl stress; S3: AMF inoculation, 350 mmol·L^−1^ NaCl stress. In this experiment, a NaCl concentration gradient of 0, 150, 250, and 350 mmol·L^−1^ was set based on the optimal salt-tolerant growth ranges of the two tested plant species (0–300 mmol·L^−1^ [27] and 0–500 mmol·L^−1^ [28] NaCl), which covered the range from non-stress to severe salt stress and allowed for a systematic comparison of their differences in salt stress responses.

Seeds were germinated in seedling trays. Uniform seedlings of similar height were transplanted into the donor and receiver chambers (three seedlings per chamber). Plants were cultivated in a greenhouse for 60 days (28 °C, 16 h light/8 h dark, 500 μmol m^−2^ s^−1^ LED, 30–50% relative humidity). Salt stress was then initiated exclusively in the donor chamber. To avoid osmotic shock, NaCl solutions were applied incrementally: 150 mL every 3 days for a total of four applications, resulting in a final cumulative volume of 600 mL. Control pots received equal volumes of distilled water. Leachate was collected in trays at the bottom of each device and recirculated. Sterile distilled water was added daily to compensate for evaporation and prevent increases in salt concentration, so as to maintain a constant system volume. Leachate electrical conductivity was monitored weekly to confirm consistent salinity levels. After 40 days of salt treatment, plants were harvested for analysis. All physiological indicators were assessed at the end of the 40-day salt stress period, using freshly collected leaves. All treatments received the same total volume of liquid (600 mL) during the salt stress period: the control and S0 groups were irrigated with distilled water, while the salt-stressed groups received NaCl solution of corresponding concentrations.

### 2.4. Measurement of Physiological Indicators: SOD, POD, MDA, SS, Pro

#### 2.4.1. Chlorophyll Content and Chlorophyll Fluorescence

Fully expanded third to fifth leaves from the apex were used. Chlorophyll content was measured in fresh leaves using a SPAD meter (TYS-4N, Mince Instrument, Xiamen, China) between 11:00–13:00. Chlorophyll fluorescence parameters were recorded with a MINI-PAM-II portable fluorometer. Following 30 min of dark adaptation, the maximum quantum efficiency of photosystem II (Fv/Fm) and non-photochemical quenching (NPQ) were determined on the same leaves.

$$NPQ=(Fm-{Fm}^{\prime })/Fm$$

Fv/Fm=(Fm−Fo)/Fm

#### 2.4.2. Antioxidant Enzyme Activities, MDA, and Osmolytes

Leaf samples (0.1 g) were homogenized in 1 mL of extraction solution to obtain a 10% homogenate. After centrifugation, the supernatants were used for the determination of enzyme activities and metabolite contents using commercial assay kits (Suzhou Grace Biotechnology Co., Ltd., Suzhou, China) according to the manufacturer’s protocols. Specifically, peroxidase (POD) activity was assayed by the guaiacol method, with absorbance monitored at 470 nm. Superoxide dismutase (SOD) activity was determined via the hydroxylamine method, and absorbance was measured at 450 nm after 30 min of dark incubation. Malondialdehyde (MDA) content was quantified by the thiobarbituric acid method, based on the absorbance values at 532 nm and 600 nm. Proline (Pro) content was analyzed using the sulfosalicylic acid method: the homogenate was heated at 90 °C for 10 min before centrifugation, and absorbance was read at 520 nm. Soluble sugar (SS) content was determined by the anthrone colorimetric method, and absorbance was measured at 620 nm after incubation at 50 °C for 20 min.

#### 2.4.3. Plant Hormones

Fresh leaf samples (0.1 g) were homogenized, and abscisic acid (ABA), salicylic acid (SA), and jasmonic acid (JA) were quantified using double-antibody sandwich ELISA kits (Shanghai KeQiao Biotechnology, Shanghai, China), following the manufacturer’s instructions. Briefly, after establishing a standard curve, homogenized samples were added to the microplate and incubated at 37 °C. After washing, a color reaction was developed in the dark and terminated with a stop solution. The absorbance (OD value) was measured at 450 nm, and hormone concentrations were calculated based on the standard curve.

### 2.5. Biomass and Nitrogen Analysis

#### 2.5.1. Biomass

At harvest (i.e., 40 days after the start of salt treatment), plants were separated into leaves, stems, and roots. Tissues were dried in a 105 °C oven for 15 min, then dried at 80 °C to constant weight. Dry weights of each part were recorded using an analytical balance. Total biomass was calculated as the sum of the dry weights of leaves, stems, and roots.

#### 2.5.2. ^15^N Labeling and Nitrogen Content

Five days before harvest (Day 95), 10 mL of 0.1 mol L^−1 15^NH_4_Cl solution (99.01 atom% ^15^N) was injected uniformly into ten equally spaced points in the donor chamber soil. After 5 days of isotope diffusion, plants were harvested. Dried plant tissues were ground to a fine powder. Total nitrogen content was determined by the Kjeldahl method. ^15^N abundance was analyzed using stable isotope ratio mass spectrometry (SIRMS) at the Institute of Botany, Chinese Academy of Sciences. Nitrogen transfer rate was calculated as the percentage of ^15^N excess recovered in the receiver plant relative to the total ^15^N excess taken up by both donor and receiver.

### 2.6. Determination of Mycorrhizal Colonization Rate

Fresh fine roots of the plant were collected and cut into root segments approximately 1 cm in length. After undergoing fixation, alkaline dissociation, acidification, and staining, the samples were mounted for observation under a microscope [2,29]. The mycorrhizal colonization rate was calculated using the cross-hatching method, with the formula as follows:

$$Mycorrhizal\;colonization\;rate=\frac{Number\;of\;infected\;root\;segments}{Total\;Number\;of\;Roots}\times 100\%$$

### 2.7. Plant Root Analysis

After harvesting the plants, the roots were carefully separated and thoroughly rinsed with deionized water to prevent salts or soil particles from compromising the scanning results. The cleaned roots were then evenly spread in a shallow layer of water within a transparent scanning tray (approximately 5 mm thick), allowing the roots to extend naturally. An Epson V700 flatbed scanner (Suwa, Japan) was used in professional mode to acquire root images, with a resolution of 400 dpi and an 8-bit grayscale image setting. The resulting digital images were analyzed using Win-RHIZO Pro 2012b software to determine root length (cm), average root diameter (mm), and number of root tips.

### 2.8. Analysis of Data

All statistical analyses were performed using SPSS 26.0. Data were first tested for normality (Shapiro–Wilk test) and homogeneity of variances (Levene’s test). Where assumptions were violated, data were transformed (e.g., log-transformation) or non-parametric tests (Kruskal–Wallis with Dunn’s post hoc) were applied. One-way ANOVA followed by Tukey’s HSD was used to compare salt treatments within each plant combination. Two-way ANOVA was used to assess the main effects of plant combination, salt concentration, and their interaction. Significance was set at *p* ≤ 0.05. Principal component analysis (PCA) was conducted in R studio 2024 with centered and scaled variables to visualize multivariate physiological patterns. Data are presented as mean ± SD or mean ± SE as indicated in figure legends.

## 3. Results

### 3.1. The Impact of Salt Stress on Mycorrhizal Colonization Rate

**Figure 2:**
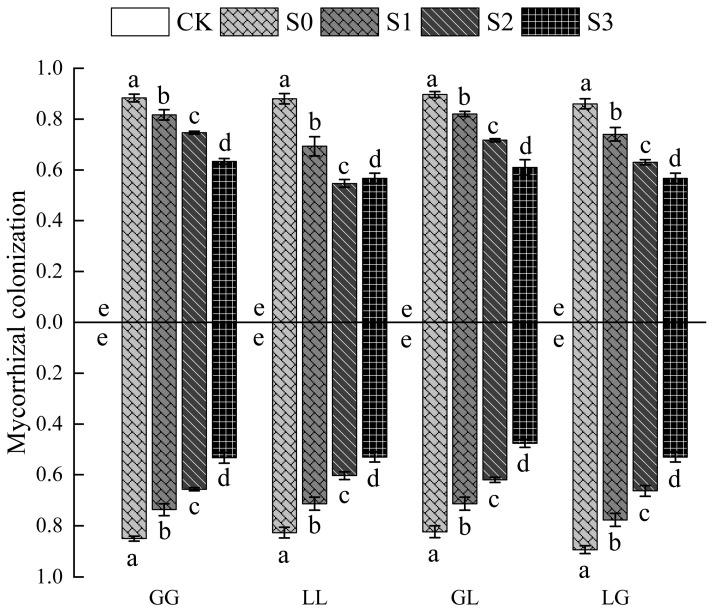
Effect of salt stress on Mycorrhizal colonization rate: Above the horizontal line was the donor; below it was the receiver. Bars with different letters (**a**–**e**) are significantly different (*p* ≤ 0.05) based on the SED mean comparisons generated from one-way ANOVA analysis in R. Error bars show ± SED.

The AMF colonization rate in the uninoculated control group (CK) was zero, confirming the validity of the inoculation experiment. Among the inoculated groups, AMF mycelium successfully penetrated the 20 μm sieve mesh. Under no salt stress (S0), the root colonization rates of both donor and recipient plants reached 86.0% to 89.7%, indicating the establishment of a uniform and highly efficient symbiotic system under these conditions. Salt stress caused a significant decrease (*p* < 0.05) in AMF colonization rates across all combinations. However, the response patterns to salt stress varied among plant combinations and their donor–recipient pairs. The G-G combination exhibited the largest reduction (49.62%), indicating the highest sensitivity of its donor roots to salt stress. In contrast, the G-L combination showed the smallest relative decrease (28.30%), demonstrating stronger salt tolerance. The L-L and L-G combinations exhibited intermediate reductions of 34.11% and 31.97%, respectively. Recipient colonization rates also decreased significantly, with the G-L combination showing the largest reduction (42.11%), while the remaining combinations exhibited reductions ranging from 37% to 41% (Figure 2).

### 3.2. The Impact of Salt Stress on Plant Biomass

**Figure 3:**
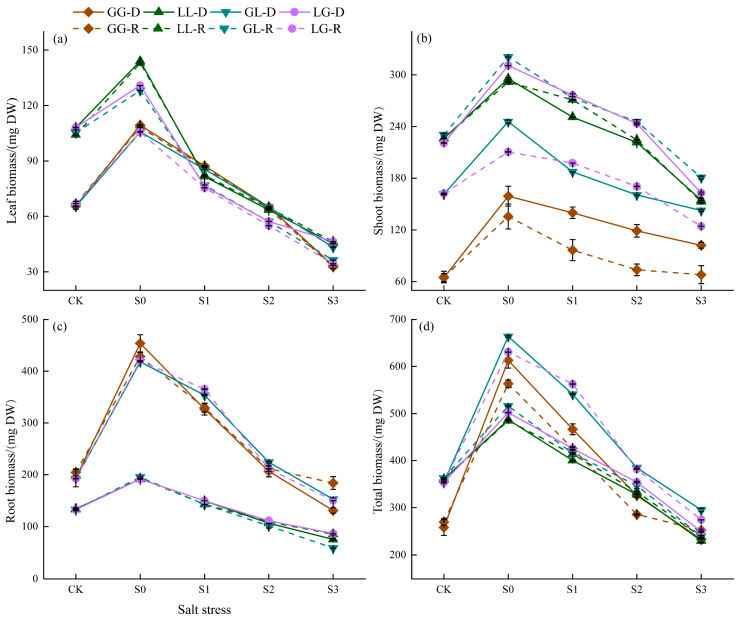
Effects of salt stress on plant biomass: respectively, the (**a**) leaf biomass, (**b**) shoot biomass, (**c**) root biomass, and (**d**) total biomass. Different colors represent different plant combinations. Solid lines indicate donors, and dashed lines indicate the receiver. GG-D denotes the donor in the G-G combination, while GG-R denotes the recipient in the G-G combination. LL-D denotes the donor in the L-L combination, while LL-R denotes the recipient in the L-L combination. GL-D denotes the donor in the G-L combination, while GL-R denotes the recipient in the G-L combination. LG-D denotes the donor in the L-G combination, while LG-R denotes the recipient in the G-G combination. Each point represents the sample mean, showing the trend of the data across different salinity gradients. The same is below.

Plant biomass responses to salt stress exhibited distinct combination-dependent and concentration-dynamic patterns (Figure 3). Overall, total biomass across all plant combinations decreased significantly with increasing salinity, though the rates and patterns of decline varied. Conspecific combinations (G-G, L-L) demonstrated greater stability, with flatter biomass decline curves, particularly at high salinity levels (S2–S3). Specifically, the recipient biomass in the GG combination exhibited the smallest decline across the salinity gradient, demonstrating optimal salt tolerance through CMNs acquisition. In contrast, heterospecific combinations (G-L, L-G) showed steeper decline curves, with the G-L recipient experiencing a sharp biomass drop at S3.

To further evaluate the combined effects of plant combinations and salinity on plant physiological and growth traits, a two-way ANOVA was performed. As shown in Table 2, salt stress exerted extremely significant effects on all measured parameters, including infection rate, biomass, root morphology, photosynthetic parameters, antioxidant enzyme activities, osmotic regulatory substances, hormone content, and nitrogen content. By contrast, plant combination treatments alone showed no significant effects on any of these indicators, suggesting that the combination treatments themselves had no substantial influence on the measured variables. However, highly significant interactions between plant combination and salinity were detected for infection rate, leaf biomass, total biomass, root morphology, SPAD value, NPQ, photosynthetic efficiency, antioxidant enzyme activities, osmotic regulators, and hormone content, whereas no significant interaction was observed for shoot biomass and root biomass. These results indicate that salinity was the dominant factor affecting all measured indices. Although plant combination treatments alone exerted no significant effects, they could modulate (alleviate or aggravate) the impacts of salt stress.

### 3.3. The Effects of Salt Stress on Plant Root Growth

**Figure 4:**
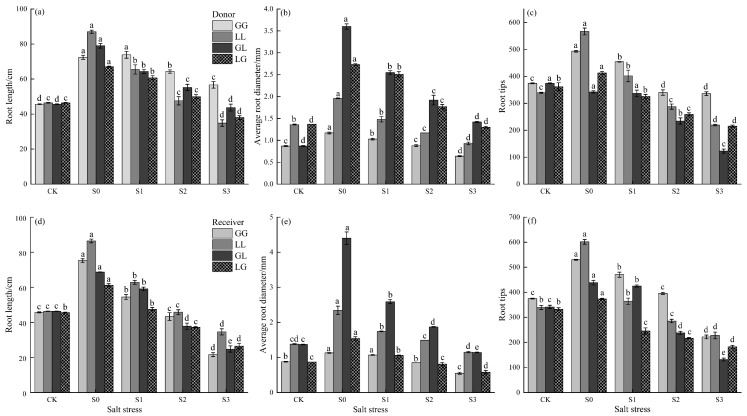
Effects of salt stress on plant root growth: (**a**–**c**) represent the donor, corresponding to root length, average root diameter, and root tips, respectively; (**d**–**f**) represent the receiver, corresponding to root length, average root diameter, and root tips. Different letters indicate significant differences among plants within the same combination under different salinity gradients (*p* < 0.05).

Salt stress significantly inhibited root growth, but root responses varied markedly among different plant combinations (Figure 4). These differences primarily manifested between homoeotic and heteroeotic combinations, as well as in the susceptibility levels of recipient plants. Specifically, the G-G combination exhibited the highest root stability, with the smallest reduction in root length and root tip number under high salinity (S3) among all combinations. The L-L combination was most sensitive to salt stress, with the greatest reduction in root length under S3 conditions. Receptor plants in heterospecific combinations suffered the most severe damage, exhibiting the lowest root length and root tip counts under high salinity, significantly lower than those of receptors in homospecific combinations.

### 3.4. Salt Stress Effects on Photosynthesis, Antioxidant Activity, and Osmotic Regulation in Different Plant Combinations

**Figure 5:**
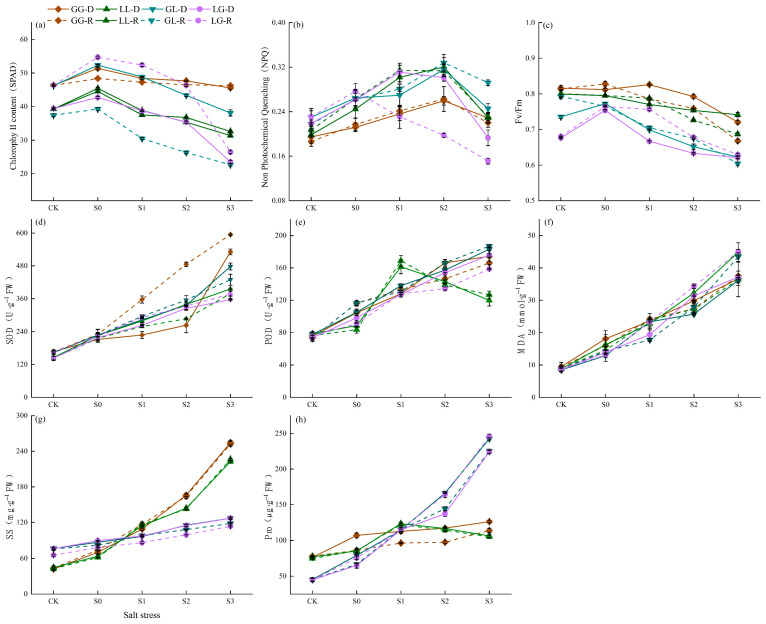
Effect of salt stress on chlorophyll fluorescence in plants: (**a**) represents chlorophyll content (SPAD), (**b**) represents Non-Photochemical Quenching (NPQ), (**c**) represents Maximum Quantum Efficiency of Photosystem II (Fv/Fm), (**d**) represents SOD activity, (**e**) represents POD activity, (**f**) represents MDA content, (**g**) represents soluble sugar (SS) content, and (**h**) represents proline (Pro) content. Different colors represent different plant combinations. Solid lines indicate donors, and dashed lines indicate the receiver. Each point represents the sample mean, showing the trend of the data across different salinity gradients.

Salt stress significantly impacts plant physiological traits, manifesting distinct response patterns particularly in photosynthetic systems, antioxidant defenses, and the accumulation of osmotic regulatory substances. Conspecific combinations maintained higher photosystem II stability under salt stress, whereas heterospecific combinations exhibited significant damage to the PSII reaction center, especially in the G-L combination (Figure 5c). NPQ values in homotic combinations increased significantly with salinity, indicating stronger photoprotection, whereas heterotic combinations exhibited slower NPQ responses with limited increases (Figure 5b). Chlorophyll content (SPAD) in homotic combinations remained elevated under salt stress, while heterotic combinations showed the most pronounced chlorophyll decline under high salinity, especially in G-L recipient plants (Figure 5a). Antioxidant enzyme activity in conspecific combinations showed stable enhancement, whereas heterospecific combinations exhibited slower enzyme activity increases. MDA accumulation significantly increased in heterozygous recipient plants, indicating weaker antioxidant capacity (Figure 5d–f). Pro and SS contents significantly increased in conspecific combinations under salt stress, while Pro showed a smaller increase in heterospecific combinations. SS content significantly rose at S3, indicating differences in osmotic regulation among plant combinations (Figure 5g,h). Data are presented as mean ± standard error. For statistical comparisons of plant combinations within each salinity treatment, see the post hoc test results in Supplementary Table S2.

### 3.5. Effects on Plant Hormones Under Salt Stress

**Figure 6:**
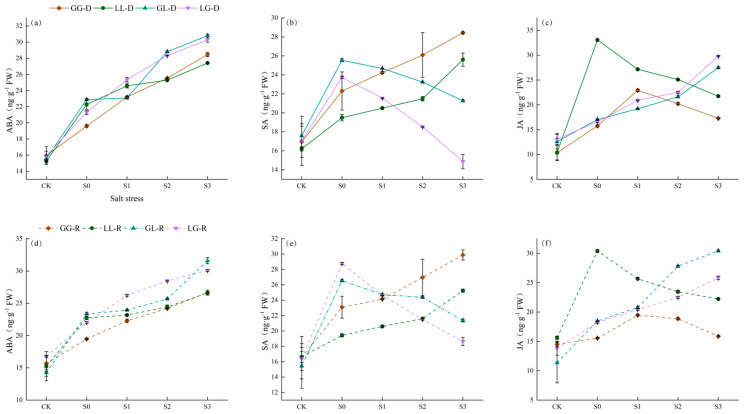
Changes in plant hormones under salt stress. (**a**) the ABA content of the donor in different combinations; (**b**) the SA content of the donor in different combinations; (**c**) the JA content of the donor in different combinations; (**d**) the ABA content of the receiver in different combinations; (**e**) the SA content of the receiver in different combinations; and (**f**) the JA content of the receiver in different combinations.

Under salt stress, endogenous hormone levels in plants undergo dramatic changes, yet homotic and heterotic combinations exhibit distinct hormonal responses; hormone content variations in the recipient plant show synchrony with the donor (Figure 6). ABA content significantly increased across all combinations with rising salinity, indicating ABA as a universal core signal for salt stress (Figure 6a,d). In conspecific combinations, jasmonic acid (JA) was strongly induced under mild to moderate salt stress (S1–S2) and exhibited synergistic accumulation with ABA (Figure 6c,f). In heterospecific combinations, JA induction was relatively weak, while salicylic acid (SA) responses were more pronounced—particularly in the LG combination, where SA peaked at S1 (Figure 6b,e).

To further explore the resource-sharing dynamics and physiological synchronization between donor and receiver plants under salt stress, paired comparisons were performed for each combination and salinity level. Data are presented as mean ± standard error. For statistical comparisons of plant combinations within each salinity treatment, see the post hoc test results in Supplementary Table S3.

### 3.6. Effects of Salt Stress on ^15^N Transfer

**Figure 7:**
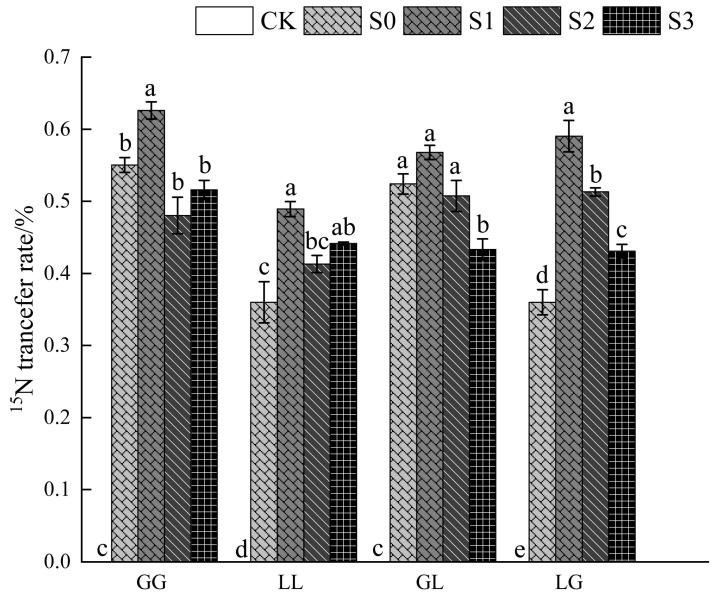
Effect of salt stress on ^15^N transfer rate: Different letters indicate comparisons within the same group under different salt stress conditions. Bar charts labeled with different letters show statistically significant differences (*p* ≤ 0.05), based on mean comparisons of standard errors generated by one-way ANOVA in SPSS software. Error bars represent ± standard error.

Under the control treatment without AMF inoculation (CK), no ^15^N was detected in any combination, confirming that N transfer was dependent on AMF inoculation. Under inoculation treatments, the S0 gradient exhibited the highest ^15^N transfer rate, with L-L, G-G, L-G, and G-L combinations reaching 48.93%, 62.61%, 59.02%, and 56.79%, respectively, indicating that under low-salinity conditions, nitrogen was efficiently transferred between plants in the presence of AMF inoculation. As salt concentration increased, the ^15^N transfer rate significantly decreased (*p* < 0.05). By the S3 treatment, the transfer rates decreased by 9.77%, 17.62%, 26.99%, and 23.70%, respectively. The most pronounced declines were observed in the L-G and G-L combinations, indicating that resource-sharing capacity in the presence of AMF is constrained in heterotypic plant combinations under high-salinity conditions, potentially affecting AMF connections. In contrast, conspecific combinations showed no significant transfer rate differences between S2 and S3 treatments, suggesting stronger CMN connection stability and higher resource homogeneity and signal consistency (Figure 7).

### 3.7. The Effect of Salt Stress on Nitrogen Content in Plants

In the donor plants, nitrogen content in all combinations under S0 treatment was significantly higher than the control (CK) (*p* < 0.05) (Table 3). The G-G combination exhibited the highest nitrogen content (4.33 ± 0.26 mg/g), representing a 21.97% increase compared to CK. As salt stress intensified, nitrogen content in all donor combinations showed a decreasing trend, with the G-G combination exhibiting the largest decline. Under S3 treatment, nitrogen content in the G-G combination dropped to 3.42 ± 0.11 mg/g, representing a 21.02% reduction compared to S0. In contrast, the nitrogen content of the L-L combination showed no significant change (*p* > 0.05) between S0 and S3 treatments, indicating strong nitrogen content stability under salt stress. In the recipient plants, the decline in nitrogen content was more pronounced (*p* < 0.05). Receptor nitrogen content in the G-G combination decreased from 4.31 ± 0.29 mg/g at S0 to 3.21 ± 0.13 mg/g at S3, a 25.52% reduction; the L-L combination showed a 25.93% decrease; the L-G combination decreased by 18.91%; and the G-L combination exhibited no significant change (*p* > 0.05).

### 3.8. PCA of Physiological and Ecological Indicators Under Different Treatments

**Figure 8:**
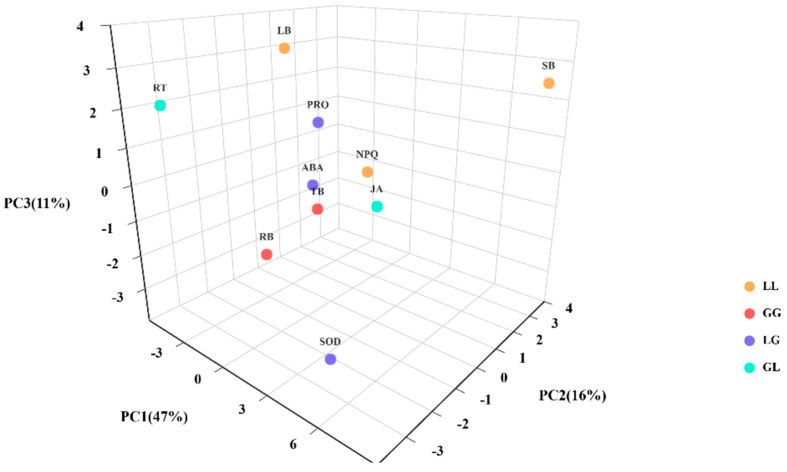
PCA analysis revealed differential responses in plant physiological and ecological indicators across different treatment groups. Different colors represent distinct plant combinations. Closer sample points indicate greater similarity in physicochemical characteristics, while greater distance signifies larger differences. Here: LB denotes leaf biomass; SB denotes stem biomass; RT indicates root tips; TB represents total biomass; RB signifies root biomass; ABA denotes abscisic acid; JA indicates jasmonic acid; SOD represents superoxide dismutase; POD denotes peroxidase.

Figure 8 presents the three-dimensional PCA analysis results for different plant combinations (G-L, L-L, L-G, G-G) based on growth and physiological indicators. PC1, PC2, and PC3 explain 47%, 16%, and 11% of the total variance, respectively. The figure reveals that different plant combinations are relatively dispersed in the three-dimensional space, indicating significant differences in their overall growth and physiological characteristics. Among these, physiological indicators such as Pro, ABA, and SOD exhibit larger projections on PC1, suggesting these indicators make important contributions to distinguishing between plant combinations. Furthermore, the relative positions of the indicators reveal potential correlations among them. For example, the proximity of Pro and ABA along the same direction may indicate synergistic effects in plant responses.

## 4. Discussion

This study systematically analyzed the physiological and hormonal responses of different plant combinations under salt stress when grown in an AMF-colonized system that permitted hyphal connections. Results indicate that plant combination types significantly influence AMF colonization, nitrogen transfer efficiency, and hormone signaling pathway activation patterns. These differences suggest that phylogenetic relatedness may affect the functionality of AMF-mediated connections. This study reveals that homoeotic plant combinations exhibit greater physiological stability under salt stress, whereas heterospecific combinations demonstrate relatively weaker responses, showing marked differences in signal synchronization and resource-sharing capabilities. This finding provides new insights into the potential functional role of AMF-mediated connectivity during salt stress and offers theoretical foundations for optimizing plant recovery strategies.

### 4.1. CMNs Stability and Phylogenetic Selection

Under salt stress conditions, AMF symbiosis significantly enhanced biomass accumulation in both donor and recipient plants, mitigating the growth suppression caused by salt stress [31,32,33,34]. The AMF colonization rate and root growth of different plant combinations exhibited combination-dependent patterns: conspecific combinations (G-G, L-L) maintained higher colonization levels even under high-salinity conditions, whereas heterospecific combinations (G-L, L-G) showed more pronounced declines in colonization rates under high-salinity treatment. This indicated that heterospecific combinations exhibited relatively lower symbiotic stability under salt stress [35,36]. For example, under S3 treatment, the colonization rate of G-L decreased by only 28.3%, lower than the 49.62% reduction observed in G-G. This difference might be related to the ability of heterospecific root exudates and microbial interactions to alleviate stress.

Root growth and total biomass changes further revealed the effect of genetic affinity: L-L exhibited a smaller biomass decline across the entire salinity gradient, demonstrating stable environmental buffering capacity; G-G showed slower initial growth but maintained better root function under high-salinity conditions; heterospecific combinations experienced the greatest total biomass decline under S3 treatment, indicating their roots were more sensitive to salt stress [37,38,39,40]. Overall, the stability of AMF-colonized associations is influenced not only by salt stress but also potentially regulated by plant phylogeny, determining the efficiency of resource sharing and signal transduction.

These findings aligned with previous studies, which indicated that kinship significantly influenced CMNs stability [41]. Prior research has shown that closely related plants exhibit greater stability within symbiotic fungal networks, effectively enhancing their adaptive capacity to environmental stresses [42]. This study further suggested that under salt stress, conspecific combinations maintained stronger physiological stability when linked by AMF than heterospecific combinations, and their root systems exhibited better functional preservation in high-salinity environments.

To further analyze the relative contributions of plant combination types and salinity to various physiological indicators, we conducted a two-way ANOVA (Table 2). Results indicated that salt stress exerted a highly significant main effect on all measured indicators (*p* < 0.001), while the main effect of plant combinations only reached significance for a few indicators (e.g., average root diameter and root tip number). Notably, the interaction between salinity and combination was significant for most indicators (e.g., mycorrhizal colonization rate, biomass, chlorophyll fluorescence parameters, antioxidant enzyme activity, hormone content, etc.) (*p* < 0.05 or *p* < 0.001). This indicates that different plant combinations adopted differentiated physiological response strategies when confronted with salinity gradients, and this variation may reflect the functional diversity of AMF-mediated connections [43].

### 4.2. Functional Group Differences in Physiological Defense Strategies

Photosystems, antioxidant systems, and osmotic regulators constitute the core defense mechanisms of plants against salt stress [30,44,45,46,47,48,49]. It has been reported that salt stress disrupted the electron transport chain, leading to impaired electron flow and excessive reactive oxygen species (ROS) production, which in turn damaged cell membrane structures [50,51]. Within photosystems, the G-G combination demonstrated higher PSII stability and NPQ regulation capacity, enabling the release of excess excitation energy through heat dissipation to reduce ROS accumulation and maintain photosynthetic performance [52,53,54]. Conversely, the G-L combination exhibited the most significant decline in Fv/Fm under high-salinity conditions, suggesting photosystem inhibition. This discrepancy might arise because the G-G combination of *Glycyrrhiza inflata* employed a photosynthetic strategy that prioritized protecting the PSII reaction center through heat dissipation, thereby preventing D1 protein degradation [55]. Leguminosae tend to maintain PSII integrity under salt stress, a strategy aligned with the energy investment required for their nitrogen-fixing symbiosis. In contrast, the desert shrub Lycium chinense in the G-L combination permits a degree of reversible PSII inactivation, redirecting resources toward osmotic regulation and secondary metabolism [56].

Regarding the antioxidant system, SOD and POD activities exhibited variations across different combinations: SOD activity showed the greatest change in the G-G combination, while POD activity increased significantly in the L-L combination. Heterospecific combinations accumulated higher MDA levels, indicating relatively weaker antioxidant capacity. This may stem from differences in the plants’ inherent salt tolerance and host functional traits [57,58,59]. The elevation of osmotic regulatory substances (Pro, SS) also exhibited combination dependency: Pro showed greater increases in conspecific combinations, while SS rose more significantly in heterospecific combinations. This might relate to genomic similarity and differences in CMNs carbon allocation strategies [60].

Plant hormones play a pivotal role in regulating physiological and biochemical processes governing normal plant growth and stress responses. Among these, abscisic acid (ABA), jasmonic acid (JA), and salicylic acid (SA) are recognized as key signaling molecules in arbuscular mycorrhizal (AM) symbiosis, exerting central regulatory functions in enhancing plant salt tolerance [61,62,63]. Regarding hormone regulation, ABA content increased across all combinations under salt stress, while SA and JA accumulation exhibited divergent trends in homoeotic versus heterospecific combinations. Conspecific combinations enhanced defense through SA synergizing with ABA, whereas heterospecific combinations responded to stress via JA synergizing with ABA [64,65,66,67,68].

We further compared hormone content differences between donor and recipient plants under the same CMN connection (Table 4) to assess signal synchrony between plants. Results showed that in conspecific combinations (e.g., G-G), differences in ABA, JA, and SA levels between donors and recipients were not significant at most salinities (*p* > 0.05), indicating efficient signal transmission and maintenance of physiological coordination. In contrast, heterospecific combinations (e.g., L-G and G-L) exhibited significant divergence in hormone levels between donors and recipients across multiple salinity treatments. For instance, in the L-G combination under S1–S3 treatments, recipient ABA and JA levels were significantly lower than donor levels (*p* < 0.05 or *p* < 0.01), indicating disrupted signal synchrony under high-salinity conditions. This may relate to reduced CMN connectivity due to differences in root exudates or carbon allocation strategies [65]. Notably, donor–recipient differences in SA were particularly pronounced in heterospecific combinations (e.g., the G-L pair showed significantly lower recipient SA than donor under S0–S2), suggesting salicylic acid-mediated defense responses may be suppressed in such combinations. These findings further confirm that plant phylogenetic relation not only influences AMF connectivity and potential resource sharing but also has the potential to profoundly influence, thereby determining the overall salt adaptation capacity of the pair [66].

### 4.3. Nitrogen Sharing and Implications for Community Assembly

Nitrogen plays a crucial role in plant growth and salt tolerance [69,70]. In this study, nitrogen transfer efficiency (potentially via AMF connections) was significantly higher in conspecific combinations than in heterospecific combinations and decreased with increasing salt stress. Under low-salinity conditions, the G-G combination exhibited the highest ^15^N transfer rate (62.61%). As salt concentration increased, transfer efficiency declined more markedly in heterospecific combinations (L-G decreased by 26.99%, G-L by 23.70%), indicating that salt stress may have weakened the connectivity and potential resource-sharing capacity between heterotypic plants in this AMF-colonized system [71,72,73]. This might result from differences in root chemical secretions and microbial interactions among plants, leading to reduced stability of their CMN networks [74].

Changes in nitrogen content between donors and recipients revealed that conspecific combinations maintained higher nitrogen stability under stress, whereas nitrogen allocation in heterospecific combinations was more dependent on plant physiological states, reflecting the selective role of CMNs in community assembly. Combined with the aforementioned physiological defense strategies, the community structure of different plant combinations may achieve differential adaptation through AMF-mediated nutrient and signal allocation, thereby influencing interspecific competition and ecological stability [75].

Combining hormone donor–receiver differences (Table 4) with ^15^N transfer rates (Figure 7) revealed that conspecific combinations (G-G) maintained high nitrogen transfer efficiency while exhibiting highly synchronized hormone levels between donors and receivers, suggesting potential synergistic effects in resource and signal transfer associated with AMF connectivity. In contrast, heterospecific combinations (L-G, G-L) exhibited not only a significant decline in nitrogen transfer rates under intensified salt stress but also a rapid widening of ABA and JA differences between donors and recipients. This suggests that signal dysregulation may further undermine the physiological basis for potential resource sharing [76]. This “signal–nutrient” decoupling phenomenon likely represents one intrinsic reason for the reduced adaptability of heterospecific combinations under high-salinity conditions.

### 4.4. PCA Reveals Differential Responses in Plant Physiological and Ecological Indicators Across Different Treatment Groups

Three-dimensional principal component analysis (PCA) further revealed combination-specific physiological functional clusters among different plant combinations under salt stress (Figure 8). The first principal component (PC1) explained 47% of the total variance, primarily driven by abscisic acid (ABA), proline (Pro), and superoxide dismutase (SOD)—key indicators central to systemic stress signaling and osmotic regulation. The L-L combination aligned closely with Pro and leaf biomass (LB) in PCA space, indicating a “growth maintenance strategy” that prioritized rapid osmotic regulation to sustain photosynthetic carbon assimilation and biomass accumulation under salt stress. Conversely, the G-G combination showed a strong association with ABA and SOD vectors, exhibiting a “defense initiation strategy” characterized by preferential activation of hormonal signaling pathways and enhanced expression of antioxidant enzyme systems.

This variation in response within the same species might be related to ecological niche succession and functional differentiation in plants. Research indicated that late-successional species (such as highly stress-tolerant perennial species) exhibited stronger dependence on AMF and significantly higher response specificity to different AMF species compared to early-successional or non-native species [77]. Subsequent studies further confirm that the establishment and growth of late-successional species strongly depend on the presence of specific AMF groups, and this “AMF dependence” is the core mechanism driving the positive feedback dynamics of plant community succession [78]. In this study, L. ruthenicum (L-L), a prototypical highly stress-tolerant, resource-efficient late-successional species, exhibits a “growth maintenance” strategy consistent with the aforementioned theory. Conversely, G. inflata (G-G), a nitrogen-fixing pioneer species, demonstrates a “defense initiation” strategy, reflecting the tendency of early-successional species to prioritize defense pathway activation under stressful conditions.

This study also found that heterospecific combinations (G-L, L-G) did not exhibit clear directional clustering in PCA space but instead showed a dispersed distribution along the PC1 and PC2 axes. This lack of functional coordination indicates that signal integration in AMF-colonized plants differs between distantly related plant partners [79]. This phenomenon may stem from interspecific variations in symbiotic compatibility. Guigard et al. investigated interactions between six rice varieties and three AMF genotypes, revealing significant variations in “symbiotic compatibility” across different variety–strain combinations. In their study, the impact on host plant dry matter accumulation ranged from –21% to +227%. This study explicitly indicates that symbiotic compatibility is the core variable determining the extent of AMF’s growth-promoting effects and induced resistance expression. Based on this, it is inferred that the heterospecific combinations (G-L, L-G) in this study likely correspond to “low-compatibility pairs,” whose CMNs linkage stability and signal transduction efficiency may be inherently weaker than those of conspecific combinations.

Through transcriptomic analysis of *Anoectochilus roxburghii* inoculated with AMF, Gu’s team discovered that AMF-mediated defense response activation relies on a regulatory network centered on ACX1 and calmodulin [80]. This network involves the coordinated expression of multiple transcription factor families, including WRKY, bHLH, ERF, NAC, and HSF. Isolated activation of a single signaling pathway is insufficient to generate systemic defense responses. The functionally discrete distribution of heterospecific combinations in PCA space in this study likely reflects the failure of these transcriptional regulatory networks to achieve synchronized activation across plants.

### 4.5. Limitations and Future Perspectives

Although this study provided insights into common mycorrhizal networks (CMNs)-mediated stress responses among plant combinations, several limitations were acknowledged. The experimental design lacked physical disruption of hyphal continuity (e.g., rotating mesh barriers), precluding unequivocal attribution of nitrogen transfer and signal transmission to CMN pathways. Alternative mechanisms, such as soil diffusion of signaling molecules, cannot be excluded. Moreover, salt stress was applied asymmetrically (donors only) to isolate transmission pathways, which, while necessary for the study aims, does not reflect uniform stress conditions in nature. Non-inoculated controls were not included across all salinity levels, limiting assessment of AMF’s direct contribution to salt tolerance. The use of a single AMF strain (*Funneliformis mosseae*) further restricted extrapolation to diverse field communities. The low biological replication (n = 3 per treatment) may reduce statistical power, and the single harvest time point precludes analysis of temporal dynamics. Additionally, the sterilized growth substrate excluded native microbial communities that might modulate plant–AMF interactions in situ. Despite these constraints, the differential responses observed between conspecific and heterospecific combinations offer valuable preliminary evidence for kinship-modulated, CMNs-associated processes under salt stress.

## 5. Conclusions

This study investigated the response patterns of different plant combinations under salt stress in an AMF-colonized system that allowed potential CMNs connections. By comparing the performance of conspecific plant combinations (e.g., G-G, L-L) with heterospecific combinations (e.g., G-L, L-G) under salt stress, the study revealed that phylogenetic affinity plays a crucial role in plant adaptability within an AMF-colonized system. The findings indicate that conspecific combinations exhibit greater physiological stability and resource sharing under high-salinity conditions when linked by AMF, suggesting that kinship may enhance the functionality of mycorrhizal networks. In contrast, heterospecific combinations exhibit weaker resource sharing and signal responses, with nitrogen transfer efficiency and physiological stability significantly declining at elevated salt concentrations.

This study further addresses the scientific questions raised in the introduction, specifically the CMNs-mediated signaling and resource-sharing mechanisms among different plant combinations under salt stress. We found that, in this system, AMF-colonized plants exhibited enhanced nitrogen transfer and salt tolerance, possibly involving plant hormone signaling pathways. This pattern is consistent with a role for mycorrhizal connections in facilitating these processes. Homologous plant combinations exhibit consistent hormone responses and resource transfer capabilities, reflecting the profound influence of plant kinship on CMNs stability and functionality.

In summary, CMNs exhibit more pronounced synergistic effects on co-planted species under salt stress, while the interactive advantages of heterocombinations are constrained under high-salinity conditions. This study not only reveals the combinatorial mechanisms underlying AMF-mediated responses to salt stress but also provides theoretical foundations and practical references for optimizing plant configuration patterns in arid and saline-alkali regions and developing microbe-based vegetation restoration strategies.

## Figures and Tables

**Table 1:** Experimental Design Treatment.

Combination	Treatment	AMF	Salt Stress
G-G	CK	-	-
	S0	+	-
	S1	+	150 mmol·L^−1^
	S2	+	250 mmol·L^−1^
	S3	+	350 mmol·L^−1^
L-L	CK	-	-
	S0	+	-
	S1	+	150 mmol·L^−1^
	S2	+	250 mmol·L^−1^
	S3	+	350 mmol·L^−1^
L-G	CK	-	-
	S0	+	-
	S1	+	150 mmol·L^−1^
	S2	+	250 mmol·L^−1^
	S3	+	350 mmol·L^−1^
G-L	CK	-	-
	S0	+	-
	S1	+	150 mmol·L^−1^
	S2	+	250 mmol·L^−1^
	S3	+	350 mmol·L^−1^

‘+’ indicated addition, while ‘-’ indicated no addition. CK referred to the treatment with no AMF inoculation, no salt stress, and physical separation that prevented hyphal penetration, thus eliminating CMN formation.

**Table 2:** Main Effects of Plant Combination and Salinity Level on Plant Variables.

Indicators	Salt Stress	Combination	Salt Stress × Combination
Mycorrhizal colonization rate	1727.786 ***	13.709 ns	2.165 *
Leaf biomass	312.247 ***	20.602 ns	6.364 ***
Shoot biomass	44.700 ***	110.766 ns	1.649 ns
Root biomass	39.548 ***	24.283 ns	1.743 ns
Total biomass	249.477 ***	14.596 ns	4.445 ***
Root length	102.047 ***	6.962 ns	1.993 *
Average root diameter	73.000 ***	78.036 ns	11.102 ***
Root tips	256.446 ***	86.504 ns	14.453 ***
SPAD	25.916 ***	22.605 ns	3.206 ***
NPQ	55.574 ***	29.262 ns	8.252 ***
FV/FM	153.282 ***	174.341 ns	7.780 ***
SOD	227.281 ***	16.079 ns	6.085 ***
POD	947.143 ***	35.626 ns	5.047 ***
MDA	551.704 ***	3.072 ns	2.804 **
SS	198.933 ***	7.384 ns	10.766 ***
Pro	3334.342 ***	15.588 ns	5.304 ***
ABA	1079.789 ***	63.298 ns	14.675 ***
SA	104.473 ***	36.728 ns	28.111 ***
JA	180.562 ***	78.292 ns	39.050 ***
N content	16.081 ***	25.959 ns	5.211 ***

F-values are followed by *p*-values. ns *p* ≥ 0.05; * *p* < 0.05; ** *p* < 0.01; *** *p* < 0.001.

**Table 3:** Effects of salt stress on plant nitrogen content.

P-P	Salt Stress	Donor (mg/g)	Receiver (mg/g)
**L-L**	CK	3.67 ± 0.10 c	3.49 ± 0.19 bc
	S0	4.28 ± 0.10 ab	4.32 ± 0.19 a
	S1	4.49 ± 0.28 a	3.80 ± 0.21 abc
	S2	4.44 ± 0.24 a	4.0 ± 0.13 ab
	S3	3.75 ± 0.11 bc	3.20 ± 0.28 c
**G-G**	CK	3.55 ± 0.20 b	3.46 ± 0.13 bc
	S0	4.33 ± 0.26 a	4.31 ± 0.29 a
	S1	3.92 ± 0.10 ab	3.94 ± 0.21 ab
	S2	3.70 ± 0.22 ab	3.51 ± 0.09 bc
	S3	3.42 ± 0.11 b	3.21 ± 0.13 c
**L-G**	CK	3.67 ± 0.10 a	3.46 ± 0.13 ab
	S0	3.22 ± 0.12 b	3.86 ± 0.25 a
	S1	3.74 ± 0.09 a	3.21 ± 0.16 b
	S2	3.66 ± 0.07 a	2.96 ± 0.12 b
	S3	3.41 ± 0.14 ab	3.13 ± 0.05 b
**G-L**	CK	3.55 ± 0.20 ab	3.49 ± 0.19 a
	S0	3.35 ± 0.10 b	3.36 ± 0.11 ab
	S1	4.00 ± 0.22 a	3.57 ± 0.22 a
	S2	3.27 ± 0.23 b	3.08 ± 0.11 b
	S3	3.39 ± 0.05 b	3.32 ± 0.00 ab

Note: Different letters indicate significant differences in the same plant combination under different salt stress conditions (*p* < 0.05). Sample mean ± SDE.

**Table 4:** The Effect of Salinity Stress on Plant Hormones. ns means *p* ≥ 0.05, * means *p* < 0.05, ** means *p* < 0.01, *** means *p* < 0.001.

Combination	Salt Stress	ABA D-R	SA D-R	JA D-R
LL	CK	0.02 ns	0.45 ns	−4.18 *
	S0	−0.47 ns	−0.78 ns	0.21 ns
	S1	1.41 *	0.09 ns	3.46 **
	S2	0.85 *	−0.87 ns	1.36 *
	S3	0.82 *	−1.44 ns	1.41 **
LG	CK	−0.87 ns	2.15 ns	1.21 ns
	S0	−0.72 ns	−1.00 *	−1.43 **
	S1	−0.88 **	−0.05 ns	−1.58 **
	S2	−0.13 ns	−1.13 **	−6.20 ***
	S3	0.23 ns	−0.09 ns	−2.94 **
GL	CK	1.34 **	0.67 ns	−0.74 ns
	S0	−0.47 **	−5.03 ***	−1.66 ***
	S1	−0.85 *	−3.11 ***	0.47 ns
	S2	3.13 **	−2.97 ***	0.01 ns
	S3	−0.80 ns	−3.76 *	3.96 **
GG	CK	0.29 ns	−0.42 ns	−5.24 *
	S0	0.15 ns	0.07 ns	2.68 **
	S1	0.92 **	−0.08 ns	1.48 **
	S2	1.33 ***	−0.12 ns	1.60 **
	S3	1.83 *	0.37 ns	−0.49 *

D-R denotes donor-receptor, and paired *t*-tests were performed under combined × salt stress conditions. Significance is indicated after the difference.

## Data Availability

The original contributions presented in this study are included in the article/Supplementary Materials. Further inquiries can be directed to the corresponding author.

## Supplementary Materials

The following supporting information can be downloaded at https://www.mdpi.com/article/10.3390/jof12040242/s1. Table S1: Post-hoc Analysis of Salt Stress Effects on Plant Biomass; Table S2: Post-Event Analysis of Physiological Data; Table S3: Post-hoc Analysis of Hormone Data.

## Abbreviations

The following abbreviations are used in this manuscript:

AMF	Arbuscular Mycorrhizal Fungi
CMNs	Common Mycorrhizal Networks
G-G	*Glycyrrhiza inflata-Glycyrrhiza inflata*
G-L	*Glycyrrhiza inflata-Lycium ruthenicum*
L-L	*Lycium ruthenicum-Lycium ruthenicum*
L-G	*Lycium ruthenicum-Glycyrrhiza inflata*
ABA	Abscisic acid
JA	Jasmonic acid
SA	Salicylic acid
POD	Peroxidase
SOD	Superoxide dismutase
MDA	Malondialdehyde
LB	Leaf biomass
SB	Shoot biomass
RT	Root tips
TB	Total biomass
RB	Root biomass
NPQ	Non-photochemical quenching
