# Supplementary material for: A Potential Role for Common Mycorrhizal Networks (CMNs) in Mediating Response Strategies and Signaling Between Different Plant Combinations Under Salt Stress
Source: J Fungi (Basel). 2026 Mar 26;12(4):242. doi: 10.3390/jof12040242 (PMC13117993; doi:10.3390/jof12040242)
Supplement: Supplementary file 1 [file jof-12-00242-s001.zip › jof-4120462-supplementary.pdf]

Supplementary Table S1 Post-hoc Analysis of Salt Stress Effects on Plant Biomass

indicators	Salt stress	Donor				Receiver			
		GG	LL	GL	LG	GG	LL	GL	LG
Leaf biomass	CK	65.83±0.39 c	108.04±0 b	66.83±1.38 c	108.16±0.07 b	66.84±1.38 c	104.11±0.47 b	106.55± 1.49 b	66.97± 1.31 c
	S0	109.4±0.15 a	144.06 b±0 a	105.73±0.02 a	130.84±0.02 a	108.27±0.12 a	143.06±0.58 a	128.12± 0.03 a	105.75± 0.13 a
	S1	87.34±0.23 b	81.55±0.04 c	85.45±0.04 b	76.27±0.79 c	86.53±0.12 b	82.22±0.32 c	76.77±0 c	75.42± 0.03 b
	S2	65.18±0.13 c	63.5±0.02 d	64.97±0.02 c	57.24±0.05 d	64.23±0.06 c	64.7±0.2 d	57.23± 0.01 d	54.94± 0.05 d
	S3	33.4±0.2 d	45.31±0.11 e	43.27±0.12 d	46.36±0.03 e	32.57±0.93 d	46.34±0.07 e	36.29± 0.01 e	34.37± 0.06 e
Shoot biomass	CK	64.55±4.83 d	226.36±0.24 c	162.65±1.28 c	220.85±0.21 d	65.18±5.56 b	226.32±0.17 c	230.62± 0.17 d	162.44± 0.68 d
	S0	159.57±10.48 a	295.52±1.15 a	244.01±1.71 a	310.67±0.15 a	135.51±14.28 a	292.01±0.56 a	320.6± 0.16 a	210.65± 0.11 a
	S1	139.89±6.91 ab	250.78±0.04 b	187.27±0.01 b	275.98±2.09 b	96.51±11.7 ab	270.95±0.5 b	273.45± 1.27 b	197.54± 0.01 b
	S2	118.94±7.43 bc	221.44±0.03 c	160.51±0.17 c	243.4±1.43 c	74.07±5.6b	224.41±0.3 c	245.51± 1.96 c	170.41± 0.07 c
	S3	102.19±3.37 c	154.53±2.08 d	142.55±0.84 d	162.46±1.07 e	68.06±9.59 b	154.12±1.43 d	182.19± 1.58 e	124.23± 0.1 e
Root biomass	CK	193.13±14.43 c	133.94±2.21 c	192.26±0.25 d	132.59±1.13 c	198.8±10.46 c	132.85±0.52 c	132.82± 1.23 c	192.61± 0.09 d
	S0	454.2±18.27 a	191.24±0.17 a	417.97±0.45 a	191.22±0.14 a	427.98±10.6 a	193.27±0.14 a	196.55± 1.28 a	420.28± 0.14 a
	S1	326.47±12.92 b	149.38±0.09 b	353.66±0.83 b	149.42±0.2 b	329.28±7.35 b	142.58±0.12 b	142.37± 0.16 b	365.29± 0.15
	S2	206.61±11.28 c	106.33±0.19 d	224.15±1.4 c	110.82±0.01 d	211.56±5.35 c	109.34±0.02 d	101.17± 3.95 d	211.6± 0.2 c
	S3	130.35±5.11 d	75.33±1.47 e	155.39±2.05 e	86.51±0 e	183.8±12.13 c	85.27±1.63 e	58.83± 0.38 e	149.51± 0.15 e
Total biomass	CK	257.68±11.61 d	360.3±2.44 c	354.91±1.29 d	353.44±1.33 c	263.98±8.9 c	359.18±0.7 c	363.45± 1.33 c	355.05± 0.7 d
	S0	613.77±9.23 a	486.76±1.28 a	661.97±2.15 a	501.89±0.24 a	563.48±20.51 a	485.28±0.53 a	517.15± 1.41 a	630.93± 0.25 a
	S1	466.35±18.03 b	400.16±0.11 b	540.94±0.83 b	425.4±2.25 b	425.79±12.16 b	413.53±0.4 b	415.82± 1.22 b	562.83± 0.15 b
	S2	325.55±14.01 c	327.77±0.19 d	384.66±1.37 c	354.22±1.43 c	285.63±3.17 c	333.75±0.29 d	346.68± 2.95 d	382.01± 0.26 c
	S3	232.54±6.05 d	229.86±2.18 e	297.94±2.39 e	248.97±1.07 d	251.86±12.59 c	239.39±1.26 e	241.02± 1.55 e	273.73± 0.11 e

Comparisons between different salinity levels within each plant combination. Results are shown as mean ± standard error. Different letters indicate significant differences. Same as below.

Supplementary Table S2 Post-Event Analysis of Physiological Data

indicators	Salt stress	Donor				Receiver			
		GG	LL	GL	LG	GG	LL	GL	LG
SPAD	CK	46.37±0.2 c	39.3±0.15 b	46.23±0.03 c	39.4±0.23 b	46.3±0.12 c	39.27±0.03 b	37.44±0.24 b	46.33±0.24 c
	S0	51.37±0.21 a	45.4±0.17 a	52.33±0.15 a	42.72±0.18 a	48.37±0.23 a	44.56±0.16 a	39.3±0.25 a	54.72±0.16 a
	S1	48.41±0.21 b	38.83±0.12 b	48.85±0.07 b	38.74±0.09 b	47.27±0.14 b	37.52±0.14 c	30.51±0.21 c	52.38±0.22 b
	S2	47.65±0.09 b	35.37±0.14 c	43.37±0.19 d	35.42±0.2 c	46.51±0.1 c	36.77±0.07 d	26.34±0.2 d	46.34±0.17 c
	S3	45.57±0.24 c	31.38±0.16 d	38.14±0.52 e	23.56±0.22 d	46.25±0.17 c	32.6±0.12 e	22.69±0.2 e	26.46±0.22 d
NPQ	CK	0.19±0.01 c	0.2±0 c	0.23±0.01 c	0.22±0 c	0.19±0 c	0.21±0.01 c	0.22±0 d	0.23±0.01 b
	S0	0.21±0 bc	0.24±0.01 b	0.26±0.01 bc	0.26±0.01 b	0.22±0.01 bc	0.26±0.01 b	0.26±0 c	0.28±0.01 a
	S1	0.24±0.01 ab	0.3±0.01 a	0.27±0.01 b	0.31±0.01 a	0.24±0 ab	0.31±0.01 a	0.28±0 bc	0.23±0.01 b
	S2	0.26±0 a	0.32±0.01 a	0.32±0.01 a	0.3±0 a	0.26±0.01 a	0.31±0.01 a	0.33±0.01 a	0.2±0 b
	S3	0.23±0 b	0.23±0.01 bc	0.25±0.01 bc	0.19±0.01 c	0.22±0 bc	0.23±0 c	0.29±0 b	0.15±0 c
Fv/Fm	CK	0.82±0 ab	0.8±0.01 a	0.74±0 b	0.68±0 b	0.81±0 b	0.8±0 a	0.79±0 a	0.68±0 c
	S0	0.81±0 b	0.8±0 a	0.77±0 a	0.75±0 a	0.83±0 a	0.8±0 ab	0.76±0 b	0.76±0 a
	S1	0.83±0 a	0.77±0 b	0.7±0 c	0.67±0 c	0.79±0 c	0.79±0 b	0.71±0 c	0.76±0 b
	S2	0.79±0 c	0.75±0 bc	0.65±0 d	0.63±0 d	0.76±0 d	0.73±0 c	0.67±0 d	0.68±0 c
	S3	0.72±0 d	0.74±0 c	0.62±0 e	0.62±0 e	0.67±0 e	0.69±0 d	0.6±0 e	0.63±0 d
SOD	CK	166.89±3.9 d	145.51±6.44 e	163.4±2.71 e	142.94±3.71 e	166.21±1.82 e	143.45±4.16 e	142.94± 3.71 e	165.8±3.34 e
	S0	211.65±6.41 c	225.41±3.32 d	227.76±11.44 d	218.22±8.35 d	230.34±3.89 d	215.66±2.97 d	226.36± 13.05 d	223.13± 7.02 d
	S1	227.7±7.3 bc	279.05±6.96 c	282.99±6.07 c	262.66±1.31 c	357.28±7.59 c	258.77±2.32 c	295.66± 3.16 c	291.52± 2.25 c
	S2	263.32±15.64 b	339.68±3.22 b	335.95±8.69 b	325.05±4.05 b	485.86±4.49 b	286.98±0.95 b	355.4±9.78 b	333.56± 1.67 b
	S3	531.24±6.11 a	395.81±1.71 a	477.82±7.22 a	356.62±0.65 a	593.9±1.33 a	378.84±5.23 a	429.54± 11.57 a	376.6±9.22 a
POD	CK	75.56±2.27 d	78.43±0.82 d	77.75±2.05 e	73.62±2.07 e	73.21±1.86 e	76.47±1.88 c	72.92±2.27 e	75.64±2.13 d
	S0	104.6±1.97 c	88.75±1.55 d	105.11±2.33 d	98.3±3.23 d	116.3±1.92 d	83.63±2.53 c	116.48± 1.71 d	91.27±2.17 c
	S1	127.95±0.59 b	119.82±3.94 c	137.94±1.35 c	127±1.75 c	133.21±1.86 c	126.69±2.68 b	130.58± 0.56 c	127.96± 0.94 b
	S2	166.24±2.26 a	142.99±1.67 b	157.25±1.76 b	154.15±1.73 b	146.52±1.32 b	138.78±1.41 b	166.21± 2.43 b	134.47± 1.07 b
	S3	174.29±0.96 a	161.37±4.91 a	183.15±1.1 a	175.84±1.27 a	166.13±1.03 a	168.8±3.87 a	186.98±0.8 a	158.66±0.3 a
MDA	CK	9.45±0.79 e	8.79±0.48 e	8.47±0.27 d	8.54±0.41 e	9.4±0.78 e	9.07±0.56 c	8.77±0.41 e	9±0.51 e
	S0	18.1±1.47 d	16.28±1.86 d	12.92±1.05 c	13.95±0.29 d	15.98±0.13 d	14.55±0.7 c	14.19±0.28 d	13.02±0.37 d

	S1	23.59±0.21 c	22.74±0.75 c	23.34±1.48 b	19.4±1.03 c	24.18±0.07 c	23.84±0.15 b	17.76±0.29 c	23.27±0.86 c
	S2	29.85±0.1 b	32.24±1.33 b	25.74±0.29 b	31.38±0.13 b	27.34±0.96 b	27.26±0.77 b	27.9±0.76 b	34.3±0.42 b
	S3	36.65±0.13 a	44.82±1.7 a	36.12±0.91 a	37.19±1.05 a	37.51±0.24 a	36.42±3.08 a	43.38±0.79 a	44.97±0.42 a
SS	CK	76.55±1.29 d	77.02±0.51 e	76.54±1.26 e	76.37±0.52 e	78.04±0.53 d	74.6±0.83 e	76.1±1.51 e	65.39±0.92 e
	S0	106.87±1.91 c	85.44±1.68 d	87.15±0.68 d	89.69±0.31 d	86.23±0.46 c	86.02±1.74 d	81.91±0.4 d	78.31±0.27 d
	S1	112.6±1.74 bc	123.39±1.18 a	96.4±0.58 c	97.22±0.39 c	96.42±0.27 b	121.91±0.94 a	98.1±1.05 c	86.5±1.03 c
	S2	117.17±0.4 b	116.59±1.79 b	115.47±0.76 b	114.92±1.32 b	97.53±1.06 b	114.24±1.64 b	107.9±0.32 b	99.18±0.12 b
	S3	126.17±0.92 a	106.23±1.48 c	127.12±1.21 a	127.01±0.48 a	113.78±1.65 a	104.99±1.16 c	118.39±0.46 a	113.62±0.56 a
Pro	CK	42.1±1.75 e	44.68±1.98 e	45.43±1.49 e	44.93±0.9 e	43.75±0.69 e	42.66±1.73 e	45.23±0.96 e	44.82±1.6 e
	S0	70.13±1.49 d	63.57±1.66 d	79.24±0.51 d	64.97±2.13 d	74.01±0.38 d	61.44±0.53 d	66.68±0.54 d	75.65±1.3 d
	S1	108.76±0.95 c	114.5±0.76 c	114.35±1.76 c	114.62±1.68 c	116.73±2.31 c	116.96±1.05 c	115.39±1.41 c	115.13±1.67 c
	S2	166±1.29 b	144.01±1.28 b	166.28±0.35 b	137.22±2.24 b	163.98±2.24 b	142.59±1.02 b	144.23±1.63 b	164.31±2.62 b
	S3	254.55±2.31 a	222.65±0.76 a	242.63±1.19 a	224.18±0.97 a	252.18±2.42 a	226.29±0.98 a	225.07±1.7 a	245.74±1.76 a

Supplementary Table S3 Post-hoc Analysis of Hormone Data

**indicators**	**Salt stress**	**Donor**				**Receiver**			
		**GG**	**LL**	**GL**	**LG**	**GG**	**LL**	**GL**	**LG**
**ABA**	CK	15.95±0.64 e	15.25±0.14 d	15.58±0.19 d	15.91±0.33 e	15.67±0.69 e	15.24±1.31 c	14.24±0.31 d	16.78±0.08 e
	S0	19.6±0.08 d	22.27±0.34 c	22.88±0.08 c	21.3±0.14 d	19.45±0.05 d	22.74±0.14 b	23.35±0.09 c	22.02±0.11 d
	S1	23.19±0.08 c	24.58±0.15 b	23.08±0.08 c	25.35±0.16 c	22.27±0.11 c	23.17±0.03 b	23.94±0.04 c	26.23±0.08 c
	S2	25.53±0.05 b	25.29±0.07 b	28.81±0.06 b	28.32±0.02 b	24.2±0.03 b	24.44±0.17 ab	25.68±0.07 b	28.45±0.07 b
	S3	28.47±0.16 a	27.41±0.04 a	30.79±0.1 a	30.3±0.18 a	26.64±0.22 a	26.59±0.1 a	31.6±0.28 a	30.08±0.09 a
**SA**	CK	16.96±0.95 c	16.22±0.13 d	17.58±0.73 d	17.05±1.51 cd	16.51±1.59 c	16.64±0.42 e	15.44±1.68 c	16.38±0.87 e
	S0	22.3±1.16 b	19.49±0.2 c	25.54±0.08 a	23.76±0.07 a	23.09±0.82 b	19.42±0.11 d	26.54±0.07 a	28.79±0.07 a
	S1	24.24±0.06 ab	20.5±0.04 bc	24.68±0.01 ab	21.54±0.03 ab	24.15±0.04 b	20.58±0.05 c	24.73±0.06 ab	24.65±0.08 b
	S2	26.09±1.36 ab	21.48±0.13 b	23.24±0.04 b	18.52±0.04 bc	26.96±1.37 ab	21.6±0.03 b	24.37±0.04 ab	21.5±0.07

	S3	28.44±0.05 a	25.61±0.4 a	21.27±0.06 c	14.86±0.44 d	29.88±0.38 a	25.24±0.11 a	21.36±0.12 b	18.62±0.3 d
**JA**	CK	10.33±0.82 d	10.38±0.93 e	12.6±0.85 e	13.11±0.66 e	14.51±0.23 d	15.62±0.15 e	11.39±2 c	13.85±0.69 e
	S0	15.76±0.1 c	33.07±0.04 a	17.04±0.04 d	16.6±0.12 d	15.54±0.05 c	30.39±0.14 a	18.47±0.07 b	18.26±0.09 d
	S1	22.93±0.13 a	27.15±0.05 b	19.2±0.05 c	20.89±0.06 c	19.47±0.07 a	25.67±0.12 b	20.78±0.08 b	20.42±0.07 c
	S2	20.23±0.12 b	25.07±0.04 c	21.61±0.11 b	22.53±0.12 b	18.87±0.1 b	23.47±0.12 c	27.81±0.07 a	22.52±0.09 b
	S3	17.28±0.07 c	21.74±0.07 d	27.47±0.08 a	29.79±0.06 a	15.88±0.05 c	22.23±0.06 d	30.42±0.08 a	25.83±0.17 a
